# Screening of the I_to_ Regulatory Subunit *Klf15* in Patients with Early-Onset Lone Atrial Fibrillation

**DOI:** 10.3389/fgene.2013.00088

**Published:** 2013-05-17

**Authors:** Morten Wagner Nielsen, Morten Salling Olesen, Lena Refsgaard, Stig Haunsø, Jesper Hastrup Svendsen

**Affiliations:** ^1^The Danish National Research Foundation Centre for Cardiac ArrhythmiaCopenhagen, Denmark; ^2^Laboratory for Molecular Cardiology, The Heart Centre, Rigshospitalet, University of CopenhagenCopenhagen, Denmark; ^3^Department of Surgery and Medicine, Faculty of Health Sciences, University of CopenhagenCopenhagen, Denmark

**Keywords:** lone AF, Klf15, ESP, genetics, mutation

## Abstract

Several studies have associated mutations in genes encoding potassium channels and accessory subunits involved in cardiac repolarization with increased susceptibility of atrial fibrillation (AF). Recently, the Krüppel-like factor 15 (*Klf15*) was found to transcriptionally control rhythmic expression of *KChIP2*, a critical subunit required for generating the transient outward potassium current (I_to_), and that deficiency or excess of Klf15 increased the susceptibility of arrhythmias. On this basis we hypothesized that mutations in *Klf15* could be associated with AF. A total of 209 unrelated Caucasian lone AF patients were screened for mutations in *Klf15* by direct sequencing. No mutations in the lone AF cohort were found. In one patient we found a synonymous variant (c.36C > T). In NHLBI GO Exome Sequencing Project (ESP) the variant was present in 31 of 4269 Caucasian individuals and in 3 of 2200 African Americans. In our cohort *Klf15* was not associated with lone AF.

## Introduction

Several studies have associated mutations in genes encoding potassium channels and accessory subunits involved in cardiac repolarization with susceptibility of atrial fibrillation (AF). The majority of mutations identified display a gain-of-function consequence on potassium currents and this, by shortening the cardiac action potential, function as a substrate for re-entry wavelets in the atria and thereby susceptibility to AF (Nattel, 2002). Gain-of-function mutations in *KCNQ1* which encodes the α-subunit of I_Ks_ (Chen et al., 2003; Hong et al., 2005; Otway et al., 2007; Das et al., 2009; Abraham et al., 2010; Bartos et al., 2011, 2013), in *KCNE1-5* which encodes the β-subunits/regulatory units of I_Ks_/I_to_ (Yang et al., 2004; Lundby et al., 2008; Ravn et al., 2008; Mann et al., 2012; Olesen et al., 2012) and in *KCND3* which encodes Kv4.3 contributing to I_to_ (Mann et al., 2012; Olesen et al., 2013) have been identified. As have mutations in *KCNH2* encoding the α-subunit of I_Kr_, in *KCNJ2* and *KCNJ8* encoding Kir2.1 and Kir6.1 respectively, in *KCNA5* encoding Kv1.5 and in *ABCC9* encoding K_ATP_ channel (Xia et al., 2005; Olson et al., 2007; Yang et al., 2009; Christophersen et al., 2012; Delaney et al., 2012; Mann et al., 2012). In a recent Nature paper by Jeyaraj et al. (2012) the Krüppel-like factor 15 (*Klf15*) was found to transcriptionally control rhythmic expression of *KChIP2*, a critical subunit required for generating I_to_ (Kuo et al., 2001), and that deficiency or excess of Klf15 increased susceptibility of ventricular arrhythmias. All these data definitely suggest that disturbances in cardiac potassium current, whether it is through mutations in α-subunits, β-subunits, or regulatory subunits, play a significant role in the pathogenesis of AF. On this basis we hypothesized that mutations in *Klf15*, because of its regulatory role of I_to_, could be associated with susceptibility of AF.

## Materials and Methods

A total of 209 patients were included from eight hospitals in the Copenhagen region of Denmark. Patient records from all in and outpatient activity in the past 10 years with the diagnosis AF were identified and read. Only lone AF patients were included in this study. ECG and clinical information was collected in order to reduce the possibility of undiagnosed heart disease. All patients were Caucasian. The study was approved by the local ethics committee (KF 01313322) and conformed to the principles outlined in the Declaration of Helsinki. Written informed consent was obtained from all patients. Gene analyses were performed using fluorescence-based real-time PCR (ABI PRISM 7900 Sequence Detection System, Applied Biosystems, CA, USA). Primers are available on request.

## Results

Clinical characteristics of the AF cohort who fulfilled the inclusion criteria are listed in Table 1. We found no mutations in *Klf15* in our AF cohort. In one patient we found a synonymous variant c.36C > T. In NHLBI GO Exome Sequencing Project (ESP) the variant was present in 31 of 4269 Caucasian individuals and in 3 of 2200 African Americans (Andreasen et al., 2013; Exome Variant Server, 2013).

**Table 1:** **Clinical characteristics of the lone AF population (*n* = 209)**.

Median age of onset, y (IQR)	31.5 (26–36)
Male gender,%	82
Height, cm	183 ± 9
Weight, kg	89 ± 17
BMI, kg/m^2^	26.7 ± 4.6
Blood pressure, mmHg
Systolic	131 ± 13
Diastolic	78 ± 9
AF type
Paroxysmal,%	55.9
Persistent,%	35.9
Permanent,%	8.2
Family history of AF
First degree relatives with AF,%	31

*All numbers are reported as mean ± standard-deviation unless otherwise noted. IQR, interquartile range*.

## Discussion

This is the first study to examine the genetic variation in *Klf15* in a lone AF cohort. *Klf15* encodes the Krüppel-like factor 15 and have been shown to elicit a regulatory role on *KChIP2* (Jeyaraj et al., 2012). KChIP2 is a critical subunit required for generating the transient outward potassium current (Kuo et al., 2001) and since several mutations in genes contributing to I_to_ have been associated with AF, it was plausible that mutations in *Klf15* could account for some AF incidents. However, we did not find any mutations in *Klf15* in our AF cohort, only a synonymous variant present in high number in ESP. In theory, synonymous variants could be pathogenic due to alterations in mRNA folding properties. However, in this case, due to the high prevalence of the found synonymous variant in ESP, this must be regarded merely as a random finding.

## Conflict of Interest Statement

The authors declare that the research was conducted in the absence of any commercial or financial relationships that could be construed as a potential conflict of interest.
